# Fluorinated Rh(I)–NHC Compounds as Potential Antibacterials Against Multidrug-Resistant *Klebsiella pneumoniae* Clinical Isolates Producing ESBL

**DOI:** 10.3390/pharmaceutics17080973

**Published:** 2025-07-28

**Authors:** Luis Ángel Turcio-García, Ricardo Parra-Unda, Hugo Valdés, Simón Hernández-Ortega, Gladymar Guadalupe Valenzuela-Ramirez, Yesmi Patricia Ahumada-Santos, Yesenia Sánchez-Lugo, Viviana Reyes-Márquez, David Morales-Morales

**Affiliations:** 1 Instituto de Química, Universidad Nacional Autónoma de México, Circuito Exterior, Ciudad Universitaria, Coyoacán, Ciudad de México C.P. 04510, Mexico; angel-_1212@hotamil.com (L.Á.T.-G.); simonho@iquimica.unam.mx (S.H.-O.); 2 Unidad de Investigaciones en Salud Pública, Facultad de Ciencias Químico Biológicas, Universidad Autónoma de Sinaloa, Culiacán C.P. 80013, Mexico; gladymarvzla.fcqb@uas.edu.mx (G.G.V.-R.); yesmiahumada@uas.edu.mx (Y.P.A.-S.); yeseniasanchez.fcqb@uas.edu.mx (Y.S.-L.); 3 Departamento de Química Orgánica y Química Inorgánica, Instituto de Investigación Química “Andrés M. del Río” (IQAR), Facultad de Farmacia, Universidad de Alcalá, Alcalá de Henares, 28805 Madrid, Spain; hugo.valdes@uah.es; 4 Departamento de Ciencias Químico-Biológicas, Universidad de Sonora, Luis Encinas y Rosales S/N, Hermosillo C.P. 83000, Mexico; viviana.reyes@unison.mx

**Keywords:** NHC–Rh(I) complexes, antibacterial activity, multidrug-resistant, *Klebsiella pneumoniae*, fluorinated complexes, metallodrugs

## Abstract

**Background/objectives:** The increasing prevalence of multidrug-resistant (MDR) bacteria, particularly *Klebsiella pneumoniae*, calls for the development of new antimicrobial agents. This study investigates a series of fluorinated azolium salts and their rhodium(I) complexes for antibacterial activity against clinical and reference strains of *K. pneumoniae*. **Methods**: Eleven fluorinated azolium salts and their corresponding Rh(I) complexes (22 compounds total) were synthesized and tested against several *K. pneumoniae* strains, including three MDR clinical isolates (U–13685, H–9871, U–13815) and ATCC reference strains. Minimum inhibitory concentrations (MICs) were determined. In silico ADMET analyses were conducted to evaluate intestinal absorption, oral bioavailability, Caco-2 permeability, carcinogenicity, solubility, and synthetic accessibility. **Results**: Among the Rh(I) complexes, **Rh–1**, **Rh–3**, and **Rh–11** showed activity against the three MDR isolates (MIC = 62.5–250 µg/mL), while **Rh–1**, **Rh–4**, **Rh–6**, and **Rh–11** were active against all ATCC strains (MIC = 3.9–250 µg/mL). The corresponding azolium salts displayed weak or no activity, highlighting the critical role of the metal center. ADMET predictions indicated that most Rh complexes had good intestinal absorption, and all except **Rh–3**, **Rh–4**, and **Rh–9** were predicted to be orally bioavailable. Compounds **Rh–1** to **Rh–7** showed Caco-2 permeability, and all were classified as non-carcinogenic. **Rh–8** to **Rh–11** exhibited lower solubility and synthetic accessibility. **Conclusions**: The results underscore the potential of fluorinated Rh(I) complexes as antibacterial agents against MDR *K. pneumoniae*, with **Rh–1** and **Rh–11** emerging as promising leads based on activity and favorable predicted pharmacokinetics.

## 1. Introduction

Multidrug-resistant (MDR) bacterial infections in hospitalized patients have become a major global concern due to the high mortality rate and increasing hospital costs [1,2,3,4]. According to the World Health Organization (WHO) and the US Centers for Disease Control and Prevention (CDC), the ever–increasing antimicrobial resistance poses an imminent threat to human health, potentially leading us back to the pre-antibiotic era. Among the bacterial pathogens, a significant number have been identified as extremely drug-resistant pathogens, including members of the ESKAPE group (*Enterococcus faecium*, *Staphylococcus aureus*, *Klebsiella pneumoniae*, *Acinetobacter baumannii*, *Pseudomonas aeruginosa*, and *Enterobacter*), which are of particular concern in relation to emerging nosocomial infections [5,6,7,8,9]. *Klebsiella pneumoniae*, for instance, is one of the most important members of the ESKAPE group. This bacterium frequently acquires and transfers resistance traits through horizontal gene transfer (HGT), enabling it to successfully evade antibiotic treatments [10,11,12]. This poses a serious problem in hospitals, as *K. pneumoniae* exhibits an extraordinary ability to colonize the skin of patients and medical staff, as well as the hospital environment. Furthermore, *K. pneumoniae* is the main cause of acute infections such as urinary tract infection (UTI), pneumonia, and wound infections [13,14]. Indeed, *K. pneumoniae* poses a significant burden on the healthcare system worldwide, serving as the main causative agent of nosocomial infections with MDR phenotypes and production of extended-spectrum β-lactamases (ESBLs). It is well known that the latter enzyme confers high resistance to certain antibiotics, such as penicillins and cephalosporins [6,8,15,16,17].

In addition to the serious health implications, infections caused by bacteria with MDR have significant economic and epidemiological consequences. Over the past two decades, these microorganisms have been involved in numerous outbreaks worldwide, with *K. pneumoniae* extended-spectrum β-lactamase producers (KPN–ESBL) being the causative agent in most cases. Studies have reported varying percentages of KPN–ESBL depending on the geographical location. For example, in some large cities in Mexico with populations of around one million, such as Monterrey, Guadalajara, and Merida, KPN–ESBL and multi-resistant strains were reported at rates of 35.9%, 26.9%, and 78.8%, respectively [18,19]. Since the introduction of antibiotics, β-lactams have been widely used as antimicrobial agents for the treatment of serious infections. However, the emergence of bacteria strains that produce ESBL and lead to MDR has severely limited the therapeutic options available to doctors [20], motivating research groups to develop and improve new antibiotics. Organometallic compounds have garnered significant attention due to their potent antimicrobial activity and pharmaceutical properties [21,22,23]. For instance, rhodium-based complexes have demonstrated the ability to disrupt the formation of antibiotic-resistant biofilms, exhibit synergistic bactericidal activity with other biocides, selectively inhibit metabolic pathways, and kill MDR bacteria. Furthermore, various mechanisms of metal-induced microbial activity have been identified, including the production of reactive oxygen species (ROS) and the depletion of antioxidants, which can cause DNA damage and inhibit enzymatic activities crucial for bacterial growth. In addition, some in vitro studies have shown that ruthenium and rhodium complexes can inhibit the activity of acetylcholinesterase and exhibit a high affinity for bovine serum albumin (BSA), transferrin, and serum. Thus, it is likely that nucleic acids and/or proteins serve as intracellular targets of these complexes in bacterial cells [24].

**Figure 1:**
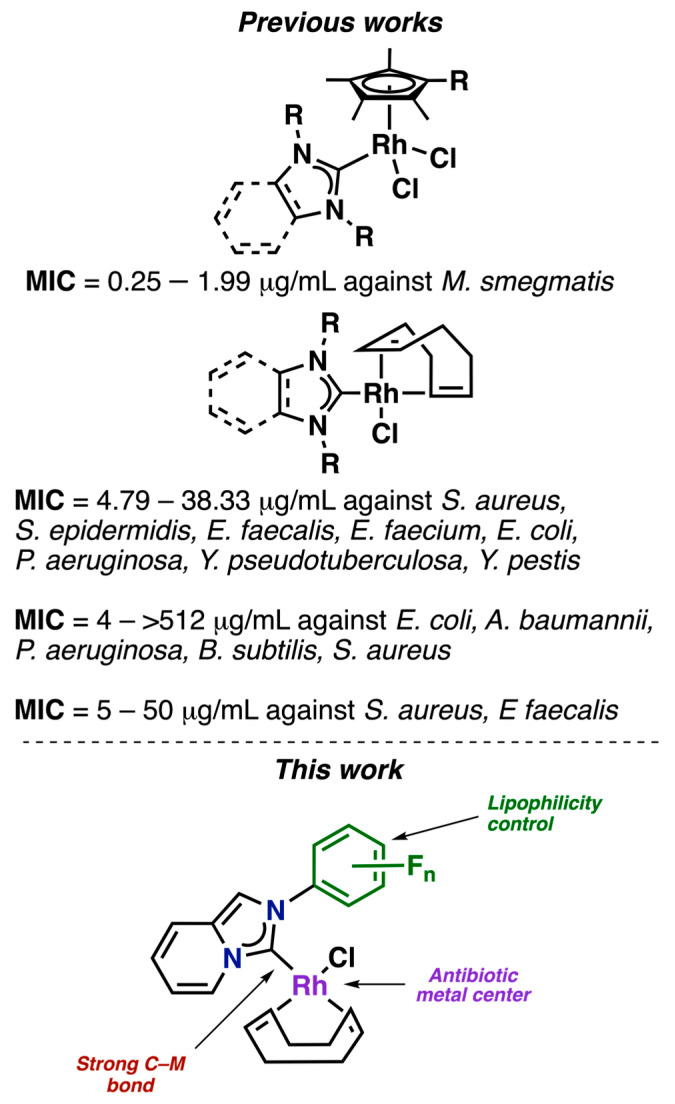
Previous works of Rh–NHC complexes with antibiotic properties [33,34,35,36]. In this work, fluorinated NHC ligands were selected for their structural versatility and ease of functionalization, allowing the introduction of diverse functionalities into the compounds.

Based on the above, we sought to evaluate a series of fluorinated compounds (Figure 1) against multi-resistant *K. pneumoniae*, which is a significant public health issue. To do so, we synthesized a family of compounds with different features. Our approach was based on previous research showing that Rhodium(I) can act as an antibacterial active center against a range of bacteria, including *E. coli*, *S. aurum*, *B. subtilis*, *B. thuringiensis*, and *P. aeruginosa* [25,26,27,28,29]. In 2019, Whitehead et al. reported that Rh(III) solutions had a MIC of 7.81 µg/mL against MDR *K. pneumoniae*. However, the antibiotic resistance of the strains was not mentioned [30]. The mechanism of action of the metal ions involves the direct contact of the metal with the bacterial cell wall and subsequent internalization in the cell, which may cause oxidation of the cellular components, generating reactive oxygen species (ROS) and disruption of the transmembrane electron transport chain [30,31]. We reasoned that the internalization of the metal center may be enhanced by the presence of a ligand. Therefore, we employed N-heterocyclic carbene (NHC) ligands, which stabilize the metal center by forming a strong M–C bond [32] and have also demonstrated significant activity against a range of bacteria, including *E. faecalis*, *E. faecium*, *S. aureus*, *S. epidermidis*, *B. subtilis*, and *M. smegmatis* [33,34,35,36]. We selected fluorinated NHC ligands for our study because of the flexibility and ease of functionalization that NHC ligands provide, allowing us to incorporate different functionalities into the compounds. Fluorine atoms were specifically incorporated, as they are known to modulate the biological activity of drugs by controlling acidity, lipophilicity, conformation, and metabolism [37,38,39,40,41]. While fluorinated NHC complexes have attracted attention for their antitumor properties [42], their use as antibiotics remains underexplored.

## 2. Results and Discussion

### 2.1. Synthesis and Characterization of the Rh(I)–NHC Complexes

**Figure 2:**
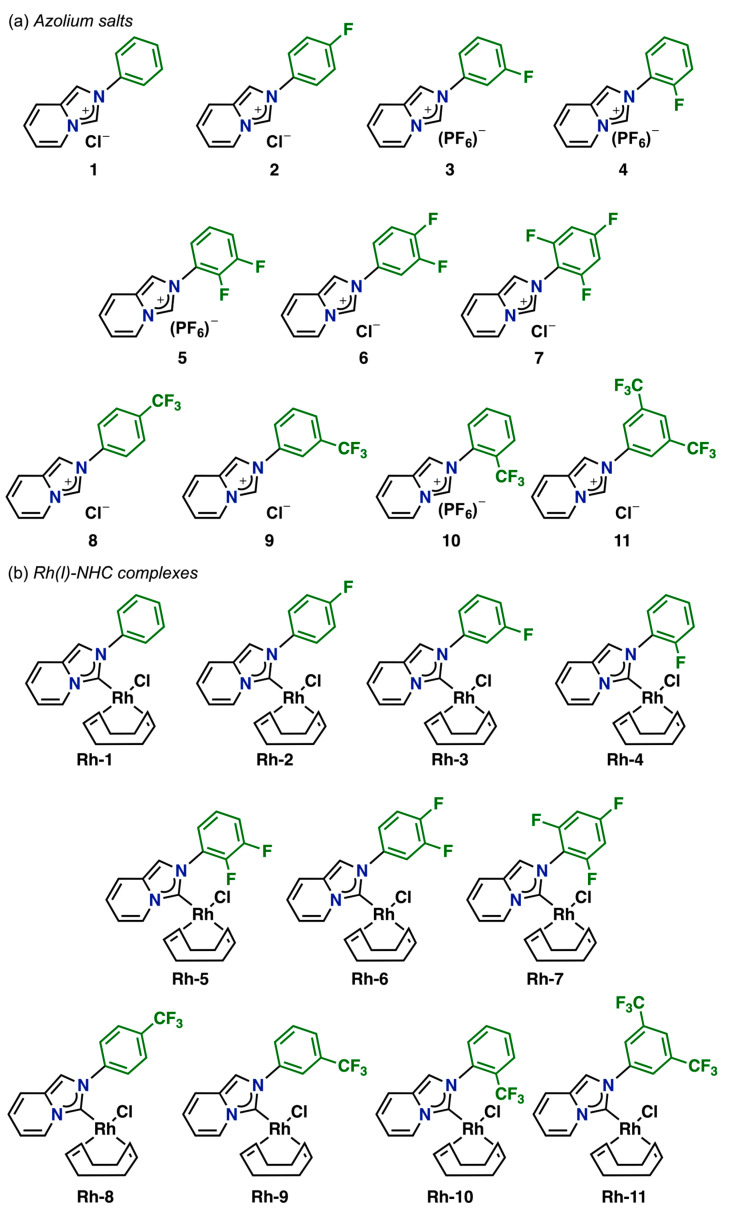
(**a**) Azolium salts and (**b**) Rh(I)–NHC complexes with fluorinated fragments.

**Figure 3:**
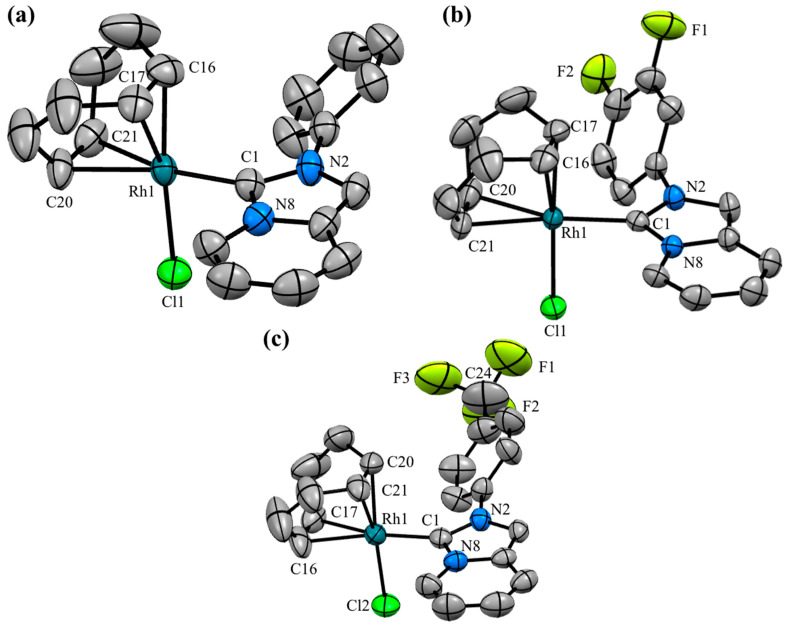
Molecular structures of (**a**) **Rh–1**, (**b**) **Rh–6,** and (**c**) **Rh–8**. Hydrogen atoms have been omitted for clarity. Ellipsoids are at the 50% probability level. Selected bond lengths (Å): **Rh–1**: Rh1–C1 2.028(3), Rh1–Cl1 2.3922(9), Rh1–C16 2.098(4), Rh1–C17 2.100(4), Rh1–C20 2.191(4), Rh1–C21 2.197(3). **Rh–6**: Rh1–C1 2.022(3), Rh1–Cl1 2.3845(9), Rh1–C16 2.118(4), Rh1–C17 2.112(4), Rh1–C20 2.199(4), Rh1–C21 2.211(4). **Rh–8**: Rh1–C1 2.020(6), Rh1–Cl2 2.389(2), Rh1–C16 2.191(4), Rh1–C17 2.211(6), Rh1–C20 2.094(6), Rh1–C21 2.118(6). Selected angles (°): **Rh–1**: C1–Rh1–Cl1: 88.68(9). **Rh–6**: C1–Rh1–Cl1: 89–41(9). **Rh–8**: C1–Rh1–Cl2: 90.1(2).

Figure 2 illustrates the fluorinated compounds evaluated in this study, which were previously described in our recent publications [43,44]. Additionally, we determined the molecular structures of three complexes (**Rh–1**, **Rh–6**, and **Rh–8**) through X–ray diffraction analyses (Figure 3). The crystals were obtained by slowly diffusing hexane into a concentrated dichloromethane solution of the respective complex. These complexes were isostructural, exhibiting coordination of the NHC ligand to the metal center and completing their coordination sphere with one COD and one chlorine ligand. The geometry around the metal was slightly distorted square–planar, with angles around the metal near 90°. The lengths between C (carbene)–Rh bonds were very similar, at approximately ~2.02 Å.

### 2.2. Isolation and Characterization of MDR Klebsiella pneumoniae

Fifteen *Klebsiella pneumoniae* isolates were obtained from different clinical samples (blood, sputum, abscesses, and urine) and characterized in selective media (i.e., MacConkey agar). All isolates were positive for urease, lysine, Simmon’s citrate, and ornithine, and negative for motility and indole production. Of the fifteen isolates, eleven were identified as MDR and ESBL producers. The MDR- and ESBL-positive (+) isolates exhibited higher resistance to all antibiotic groups than the non-MDR and ESBL-negative (–) isolates, as expected.

The antibiotic susceptibility test results revealed that eleven isolates were resistant to multiple drugs, while four isolates were exclusively resistant to Ampicillin. Among the multiple drug-resistant isolates, the highest frequency of resistance was observed for β-lactams, with 55.3%, followed by tetracyclines (50.0%), sulfonamides (46.7%), furans (46.7%), quinolones (43.3%), and aminoglycosides (28.9%).

### 2.3. Antibacterial Activity Evaluation (MIC/MBC)

In our initial experiments, we assessed the efficacy of the 22 prepared compounds by determining their minimum inhibitory concentration (MIC) and minimum bactericidal concentration (MBC) against six different control strains, including *Staphylococcus aureus* mecA (–) (ATCC 25923), *Staphylococcus aureus* mecA (–) (ATCC 29213), *Staphylococcus aureus* mecA (+) (ATCC 43300), *Escherichia coli* (ATCC 25922), *Enterococcus faecalis* (ATCC 29212), and ESBL *Klebsiella pneumoniae* (ATCC 700603), as well as an isolate of *Streptococcus* spp. obtained from nasopharyngeal exudate. Our findings, summarized in Table 1, revealed that the azolium salts exhibited very low activity against all the strains, with MIC/MBC > 250 μg/mL. However, the presence of the metal fragment resulted in significantly lower values. For instance, complex **Rh–1** displayed MIC/MBC values of 62.5/62.5 μg/mL against *S. aureus* (ATCC 29213) and *E. faecalis* (ATCC 29212). Intriguingly, the presence of a single fluorine atom did not impact the activity of the complexes, whereas the presence of two fluorine atoms at positions 3 and 4 (**Rh–6**) led to a decrease in MIC/MBC concentration to 62.5 μg/mL against *S. aureus* (ATCC 25923), *S. aureus* (ATCC 43300), and *Streptococcus* spp. In comparison, the complex with three fluorine atoms (**Rh–7**) was highly selective against *S. aureus* (ATCC 29213), exhibiting a MIC of 3.9 μg/mL. Notably, complex **Rh–11**, which contains two –CF_3_ groups, displayed the lowest MIC/MBC concentrations, ranging from 0.97 to 62.5 μg/mL, depending on the strain, with the lowest values observed against *E. faecalis* (ATCC 29212) (MIC/MBC = 0.97/15.6 μg/mL). These values are similar to those obtained for commonly used antibiotics such as linezolid (LIN), gentamicin (GEN), or amoxicillin (AMOX).

To develop new antibiotics against MDR and ESBL-producing *Klebsiella pneumoniae*, we assessed the MIC and MBC values for all fluorinated compounds against our clinical *K. pneumoniae* isolates: U–13685, H–9871, U–13815, H–9866, H–166, and S–401 (Table 2). Azolium salts were inactive against all strains (MIC/MBC >250/250 µg/mL). However, complexes **Rh–1**, **Rh–3**, **Rh–4**, **Rh–5**, **Rh–6,** and **Rh–11** exhibited activity against isolates U–13685, H–9871, U–13815, and H–9866, with MIC values ranging from 62.5 to 250 µg/mL. Moreover, most of these complexes were bactericidal against these strains, except for **Rh–1** and **Rh–6**, which showed a bacteriostatic effect against isolate U–13815. However, these complexes exhibited MIC values >250 µg/mL against clinical isolates H–9866, H–166, and S–401. Interestingly, complex **Rh–3**, containing a fluorine atom at the 3-position, displayed the most potent activity against our clinical isolates, with the lowest MIC/MBC values (62.5 µg/mL).

The results described above are consistent with the fact that Gram-positive bacteria have a simpler cell wall structure, which facilitates the transfer of the complexes inside the cells. In contrast, Gram-negative bacteria have an outer lipopolysaccharide membrane that makes it more difficult for the complexes to penetrate the cell wall. However, it is interesting to note that despite this barrier, complexes **Rh–1**, **Rh–3**, **Rh–4**, **Rh–5**, **Rh–6**, and **Rh–11** were still active against isolates U–13685, H–9871, U–13815, and H–9866. This suggests that the different resistance phenotypes of these isolates may play a role in the ability of the complexes to penetrate the cell wall.

### 2.4. Time–Killing Kinetics of Rh(I)–NHC Complexes

**Figure 4:**
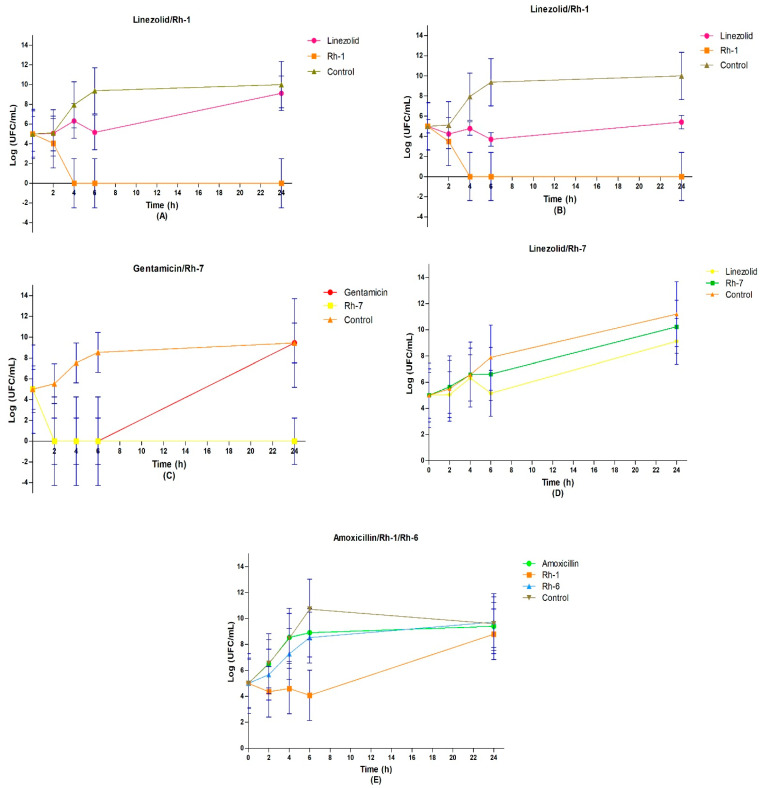
Time–kill curves of: (**A**) *S. aureus* (ATCC 29213) treated with **Rh–1** at 1 X MIC ** and (**B**) 4 X MIC *; (**C**) *E. coli* (ATCC 25922) and (**D**) *S. aureus* (ATCC 29213) treated with **Rh–7** at 1 X MIC *; (**E**) *K. pneumoniae* U–13815 (clinical isolate) treated with **Rh–1** and **Rh–6** at 1 X MIC. All the time–kill curves were recorded during 24 h. The blue bars in each value indicate the standard deviation of the mean. One-way ANOVA followed by Tukey’s multiple comparisons test was applied. * *p* < 0.05, ** *p* < 0.01. (**A**) (linezolid vs. Rh-1 (1 MIC)) *, (**A**) (Rh-1 vs. control (1 MIC)) **, (**B**) (Rh-1 vs. control (4 MIC)) * and (**C**) (Rh-7 vs. control) *. The control compounds are described in the Supplementary Information.

To determine the bactericidal kinetics of the organometallic compounds, we utilized the broth microdilution method. We focused on those complexes that exhibited the lowest MIC values, which were previously determined using the broth microdilution method. As shown in Figure 4, complex **Rh–1** demonstrated a concentration-dependent bactericidal effect at 1X MIC and 4X MIC (Figure 4A,B). Specifically, **Rh–1** showed the same germicidal speed against *S. aureus* (ATCC 29213) at concentrations of 1X MIC (62.5 µg/mL) and 4X MIC (250 µg/mL) after 4 h (5 log10 CFU/mL). Furthermore, **Rh–1** exhibited a bacteriostatic effect against the clinical isolate of *Klebsiella pneumoniae* (U–13815), reducing bacterial growth (1 log10 CFU/mL) during the initial 6 h at a concentration of 1X MIC (250 µg/mL) (Figure 4E).

Compound **Rh–7** displayed remarkable bactericidal activity against *E. coli* (ATCC 25922) at 1 MIC after only 2 h of incubation, reducing the bacterial load by 5 log10 CFU/mL (Figure 4D). Conversely, it exhibited a bacteriostatic effect against *S. aureus* (ATCC 29213) at 1 MIC, causing a difference of 1 log10 CFU/mL until 6 h of incubation (Figure 4D). The growth index of *S. aureus* (ATCC 29213) remained unchanged between long incubation times (6–24 h) and short times, exhibiting a growth rate of 3 log10 CFU/mL until 6 h (Figure 4D). Interestingly, gentamicin demonstrated a similar effect to **Rh–7** in the first 6 h, reducing the bacterial load by 5 log10 CFU/mL, but the growth of *E. coli* (ATCC 25922) increased considerably afterward, matching the growth control at 24 h at 1 MIC. As shown in Figure 4D, the growth rate of *E. coli* (ATCC 25922) treated with 1 MIC was higher in shorter incubation times (0–6 h), reaching 3–4 log10 CFU/mL, whereas in longer times, it reduced to 1 log10 CFU/mL from 6 to 24 h. Conversely, linezolid exhibited a bacteriostatic effect, reducing the bacterial load by 1 log10 CFU/mL at 1X MIC (1 µg/mL) and by 2 log10 CFU/mL at 4X MIC (4 µg/mL).

Complex **Rh–6** showed a similar effect on bacterial growth (3 log10 CFU/mL) to that of amoxicillin against the clinical isolate of *K. pneumoniae* (U–13815). Specifically, the growth rate of *K. pneumoniae* (U–13815) was 6 log10 CFU/mL during the 0–6 h incubation period and 0 log10 CFU/mL during the 6–24 h period (Figure 4E). Moreover, there was an observed decline in the growth of U–13815 of approximately 1 log10 CFU/mL at 24 h in the growth control (250 µg/mL). Notably, a noticeable difference in bacterial presence was observed after 24 h following contact with **Rh–1** and **Rh–6** in *K. pneumoniae* (U–13815), compared to amoxicillin, using the Gram stain technique (Figure 4E).

### 2.5. ADMET Prediction/Pharmacokinetic Parameters

We performed ADMET (Absorption, Distribution, Metabolism, Excretion, and Toxicity) predictions for the azolium salts and complexes. Zolpidem and cisplatin were included as reference drugs for comparative purposes (Table 3). The ADMET profile of a compound is a critical determinant of its pharmacological efficacy and potential toxicity. Predictions were carried out using the admetSAR and SwissADME online platforms [45,46]. The results indicate that all the azolium salts are orally bioavailable in humans and can cross the blood–brain barrier and Caco-2 cells. Both the azolium salts and the reference drugs fall under category III of acute oral toxicity and are not considered carcinogenic. Additionally, compounds **1**, **2**, **3**, **6**, **8**, **9**, **10**, and **11** do not exhibit hepatotoxicity, while compounds **1**, **2**, **5**, **6**, **7**, **8**, **9**, **10**, and **11** show intestinal absorption in humans.

The ADMET analysis predicted that all the metal complexes in the study were permeable to the blood–brain barrier. Additionally, Complexes **Rh–1**, **Rh–2**, **Rh–3**, **Rh–4**, **Rh–6**, **Rh–7**, **Rh–8**, and **Rh–10** showed intestinal absorption, similar to cisplatin. Most of the Rh(I)–NHC complexes and cisplatin were found to be orally bioavailable in humans, with the exception of **Rh–3**, **Rh–4**, and **Rh–9**. Interestingly, all the complexes and drugs studied were classified as having acute oral toxicity category III and were found to be non-carcinogenic, with the exception of cisplatin. Furthermore, Rh(I)–NHC complexes (excluding **Rh–5**) were found to be non-mutagenic and non-hepatotoxic. Moreover, complexes **Rh–1** to **Rh–7** were found to be permeable to Caco–2 cells, as was cisplatin. However, complexes **Rh–8** to **Rh–11** were not permeable to Caco–2 cells. All organometallic compounds and cisplatin were found to produce aquatic toxicity in fish and crustaceans, but they did not show similarities with lead.

According to the SILICOS–IT model, the solubility of all Rh(I)–NHC complexes in water is categorized as moderately to slightly soluble. However, most of the compounds comply with the chemical rules of “LIPINSKI” and are similar to commercial drugs, with the exception of **Rh–8**, **Rh–9**, **Rh–10**, and **Rh–11**. These compounds were predicted to have a low synthetic accessibility, which means that they are more difficult to synthesize than the other complexes. On the other hand, the rest of the Rh(I)–NHC complexes have a synthetic accessibility close to 1, indicating that they are very easy to synthesize.

## 3. Conclusions

We investigated the antibiotic activity of a series of fluorinated compounds against various Gram-positive and -negative bacteria. While the azolium salts displayed minimal activity against the tested strains, the Rh(I) complexes demonstrated highly promising results. Notably, the complex containing two-CF_3_ groups displayed a remarkable MIC value of up to 0.97 μg/mL against *E. faecalis* (ATCC 29212). Additionally, we observed that the presence of a single fluorine atom in the complexes produced an active species that exhibited comparable antibacterial activity to amoxicillin when tested against our clinical isolates of *Klebsiella pneumoniae*. These encouraging results suggest that the Rh(I) complexes could serve as useful models for the development of novel antibacterial drugs. Other potential biological applications using these complexes are currently under study in our laboratories.

## Figures and Tables

**Table 1:** Minimal Inhibitory Concentration (MIC) and Minimal Bactericidal Concentration (MBC) for fluorinated compounds against ATCC strains.

Compound	MIC/MBC (µg/mL)						
	ATCC 25923	ATCC 29213	ATCC 43300	ATCC 25922	ATCC 29212	ATCC 700603	*Streptococcus* spp.
**1**	NT	>250/>250	NT	>250/>250	NT	>250/>250	NT
**2**							
**3**							
**4**							
**5**							
**6**							
**7**							
**8**	>250/>250	250/250	>250/>250		>250/>250		>250/>250
**9**	NT	>250/>250	NT		NT		NT
**10**							
**11**							
**Rh–1**	250/250	62.5/62.5	250/250	250/250	62.5/62.5		250/250
**Rh–2**	NT	>250/>250	NT	>250/>250	NT		NT
**Rh–3**	250/250	250/250	250/250		>250/>250		>250/>250
**Rh–4**				250/>250	250/250		250/250
**Rh–5**	62.5/62.5			>250/>250	62.5/62.5		15.6/15.6
**Rh–6**	62.5/250		62.5/62.5	250/>250			62.5/62.5
**Rh–7**	NT	3.9/15.6	NT	250/250	NT		NT
**Rh–8**		>250/>250	>250/>250	>250			
**Rh–9**	62.5/62.5	250/250	250/250		62.5/62.5		62.5/62.5
**Rh–10**							15.6/62.5
**Rh–11**	15.6/15.6		62.5/62.5	250/>250	0.97/15.6		15.6/15.6
**LIN**	4/>32	1/8	4/>32	>32/>32	2/>32	NT	1/8
**GEN**	1/1	1/1	>32/>32	0.25/1	4/16	0.5/0.5	<0.125/<0.125
**AMOX**	<4/<4	<4/<4	32/32	<4/<4	4/4	>256/>256	32/32

**NT:** not evaluated. **ATCC 25923**: *Staphylococcus aureus mecA* (–), susceptible to methicillin; **ATCC 29213**: *Staphylococcus aureus mecA* (–), susceptible to oxacillin; **ATCC 43300**: *Staphylococcus aureus mecA* (+), resistant to methicillin; **ATCC 25922**: Susceptible *Escherichia coli*; **ATCC 29212**: *Enterococcus faecalis* susceptible to vancomycin; **ATCC 700603**: ESβL (extended-spectrum β-lactamases) *Klebsiella pneumoniae*. ***Streptococcus* spp**.: isolated from nasopharyngeal exudate. **LIN:** linezolid, **GEN:** gentamicin, **AMOX:** amoxicillin.

**Table 2:** Minimal Inhibitory Concentration and Minimal Bactericidal Concentration (µg/mL) for fluorinated compounds against isolates of MDR *Klebsiella pneumoniae*.

Compound	MIC/MBC (µg/mL)					
	U–13685	H–9871	U–13815	H–9866	H–166	S–401
**1**	>250/>250					
**2**						
**3**						
**4**						
**5**						
**6**						
**7**						
**8**						
**9**						
**10**						
**11**						
**Rh–1**	250/250	250/250	250/250	>250/>250	>250/>250	>250/>250
**Rh–2**	>250/>250	>250/>250	>250/>250			
**Rh–3**	250/250	250/250	62.5/62.5			
**Rh–4**	>250/>250	>250/>250	250/250			
**Rh–5**				250/250		
**Rh–6**			250/>250	>250/>250		
**Rh–7**			>250/>250			
**Rh–8**						
**Rh–9**						
**Rh–10**						
**Rh–11**	250/250		250/>250	250/250		
**GEN**	<0.125/<0.125	1/1	1/1	<0.125/<0.125	0.5/0.5	<0.125/<0.125
**AMOX**	64/64	>256/>256	>256/>256	128/128	>256/>256	>256/>256

**GEN:** gentamicin, **AMOX:** amoxicillin.

**Table 3:** List of ADMET properties for the fluorinated compounds.

MODEL	Azolium Salts											Rh(I)–NHC Complexes											ZP	CP
	1	2	3	4	5	6	7	8	9	10	11	Rh–1	Rh–2	Rh–3	Rh–4	Rh–5	Rh–6	Rh–7	Rh–8	Rh–9	Rh–10	Rh–11		
**ADMETsaR**																								
**Human intestinal** **absorption**	+	+	–	–	+	+	+	+	+	+	+	+	–	+	+	–	+	+	+	–	+	–	+	+
**Human oral** **bioavailability**	+	+	+	+	+	+	+	+	+	+	+	+	+	–	–	+	+	+	+	–	+	+	+	+
**Blood–brain** **barrier**	+	+	+	+	+	+	+	+	+	+	+	+	+	+	+	+	+	+	+	+	+	+	+	+
**Mutagenesis (AMES)**	+	+	+	+	+	+	+	–	–	+	–	–	–	–	–	–	–	–	–	–	–	–	+	–
**Acute oral toxicity** **(c) kg/mol**	(III)	(III)	(III)	(III)	(III)	(III)	(III)	(III)	(III)	(III)	(III)	(III)	(III)	(III)	(III)	(III)	(III)	(III)	(III)	(III)	(III)	(III)	(III)	(III)
**Permeability/** **Caco–2**	+	+	+	+	+	+	+	+	+	+	+	+	+	+	+	+	+	+	–	–	–	–	+	+
**Carcinogenicity**	–	–	–	–	–	–	–	–	–	–	–	–	–	–	–	–	–	–	–	–	–	–	–	+
**Hepatotoxicity**	–	–	–	+	+	–	+	–	–	–	–	–	–	–	–	+	–	–	–	–	–	–	+	–
**Fish aquatic** **toxicity**	+	+	+	+	+	+	+	+	+	+	+	+	+	+	+	+	+	+	+	+	+	+	–	+
**Crustacean aquatic** **toxicity**	+	+	+	+	+	+	+	+	+	+	+	+	+	+	+	+	+	+	+	+	+	+	–	+
**SwissADME**																								
**Solubility in water (Log S)** **SILICOS–IT (–)**	3.78	4.07	4.07	4.07	4.35	4.35	4.62	4.67	4.67	4.67	5.53	6.10	6.74	6.74	6.74	7.0	7.0	7.26	7.29	7.29	7.29	8.10	5.0	2.1
**Gastrointestinal absorption**	+	+	–	–	–	+	+	+	+	+	+	+	–	–	–	–	–	–	–	–	–	–	+	+
**Similarity to drugs “LIPINSKI”**	+	+	+	+	+	+	+	+	+	+	+	+	+	+	+	+	+	+	–	–	–	–	+	+
**Synthetic accessibility**	1.77	1.81	2.19	2.11	2.25	1.97	1.90	1.86	1.94	2.30	2.16	3.56	4.09	4.10	4.12	4.13	4.10	4.12	4.14	4.18	4.24	4.33	2.67	/
**Lead resemblance**	–	–	–	–	–	–	–	–	–	–	–	–	–	–	–	–	–	–	–	–	–	–	–	–

Note: “+”, predicted presence of the property; “–”, predicted absence of the property.

## Data Availability

The data that support the findings of this study are available in the Supplementary Materials of this article.

## Supplementary Materials

The following supporting information can be downloaded at: https://www.mdpi.com/article/10.3390/pharmaceutics17080973/s1, Supplementary material includes, full descriptions of the materials and methods employed for the isolation and characterization of *Klebsiella pneumoniae*, the antibacterial activity evaluation (MIC/MBC), the time–killing kinetics of Rh(I)–NHC complexes and the ADMET prediction/Pharmacokinetic parameters. As well as full description of the data collection and refinement as well as crystallographic data for compounds Rh–1 (CCDC 2280627), Rh–6 (CCDC 2280628) and Rh–8 (CCDC 2280629). The later been deposited at the Cambridge Crystallographic Data Centre. Copies of this information are available free of charge on request from The Director, CCDC, 12 Union Road, Cambridge, CB2 1EZ, UK (Fax: +44-1223-336033; e-mail deposit@ccdc.cam.ac.uk or www: http://www.ccdc.cam.ac.uk (accessed on 1 June 2025)).

## References

[1] van Duin D., Paterson D.L. (2016). Multidrug-Resistant Bacteria in the Community: Trends and Lessons Learned. Infect. Dis. Clin. N. Am..

[2] Spellberg B., Blaser M., Guidos R.J., Boucher H.W., Bradley J.S., Eisenstein B.I., Gerding D., Lynfield R., Reller L.B., Rex J. (2011). Combating antimicrobial resistance: Policy recommendations to save lives. Clin. Infect. Dis..

[3] Rodriguez-Villodres A., Martin-Gandul C., Penalva G., Guisado-Gil A.B., Crespo-Rivas J.C., Pachon-Ibanez M.E., Lepe J.A., Cisneros J.M. (2021). Prevalence and Risk Factors for Multidrug-Resistant Organisms Colonization in Long-Term Care Facilities Around the World: A Review. Antibiotics.

[4] Medina E., Pieper D.H. (2016). Tackling Threats and Future Problems of Multidrug-Resistant Bacteria. Curr. Top. Microbiol. Immunol..

[5] Tigabu A., Getaneh A. (2021). Staphylococcus aureus, ESKAPE Bacteria Challenging Current Health Care and Community Settings: A Literature Review. Clin. Lab..

[6] Santajit S., Indrawattana N. (2016). Mechanisms of Antimicrobial Resistance in ESKAPE Pathogens. Biomed. Res. Int..

[7] Rice L.B. (2008). Federal funding for the study of antimicrobial resistance in nosocomial pathogens: No ESKAPE. J. Infect. Dis..

[8] Bush K., Jacoby G.A. (2010). Updated functional classification of beta-lactamases. Antimicrob. Agents Chemother..

[9] Rice L.B. (2010). Progress and challenges in implementing the research on ESKAPE pathogens. Infect. Control Hosp. Epidemiol..

[10] Tacconelli E., Cataldo M.A., Dancer S.J., De Angelis G., Falcone M., Frank U., Kahlmeter G., Pan A., Petrosillo N., Rodriguez-Bano J. (2014). ESCMID guidelines for the management of the infection control measures to reduce transmission of multidrug-resistant Gram-negative bacteria in hospitalized patients. Clin. Microbiol. Infect..

[11] CDC (2019). Antibiotic Resistance Threats in the United States, 2019.

[12] Jacoby G.A. (2009). AmpC beta-lactamases. Clin. Microbiol. Rev..

[13] Gonzalez A.C., Nieves B., Solorzano M., Cruz J., Puig J., Moreno M. (2013). Characterization of extended-spectrum beta-lactamases-producing *Klebsiella pneumoniae* isolates of two intensive care units. Rev. Chilena Infectol..

[14] Ho J., Tambyah P.A., Paterson D.L. (2010). Multiresistant Gram-negative infections: A global perspective. Curr. Opin. Infect. Dis..

[15] Zhao W.H., Hu Z.Q. (2013). Epidemiology and genetics of CTX-M extended-spectrum beta-lactamases in Gram-negative bacteria. Crit. Rev. Microbiol..

[16] Bonnet R. (2004). Growing group of extended-spectrum beta-lactamases: The CTX-M enzymes. Antimicrob. Agents Chemother..

[17] Babic M., Hujer A.M., Bonomo R.A. (2006). What’s new in antibiotic resistance? Focus on beta-lactamases. Drug. Resist. Updat..

[18] Uc-Cachon A.H., Gracida-Osorno C., Luna-Chi I.G., Jimenez-Guillermo J.G., Molina-Salinas G.M. (2019). High Prevalence of Antimicrobial Resistance Among Gram-Negative Isolated Bacilli in Intensive Care Units at a Tertiary-Care Hospital in Yucatan Mexico. Medicina.

[19] de Kraker M.E., Stewardson A.J., Harbarth S. (2016). Will 10 Million People Die a Year due to Antimicrobial Resistance by 2050?. PLoS Med..

[20] Durdu B., Meric Koc M., Hakyemez I.N., Akkoyunlu Y., Daskaya H., Sumbul Gultepe B., Aslan T. (2019). Risk Factors Affecting Patterns of Antibiotic Resistance and Treatment Efficacy in Extreme Drug Resistance in Intensive Care Unit-Acquired *Klebsiella pneumoniae* Infections: A 5-Year Analysis. Med. Sci. Monit..

[21] Frei A. (2020). Metal Complexes, an Untapped Source of Antibiotic Potential?. Antibiotics.

[22] Liang J., Sun D., Yang Y., Li M., Li H., Chen L. (2021). Discovery of metal-based complexes as promising antimicrobial agents. Eur. J. Med. Chem..

[23] Patra M., Gasser G., Metzler-Nolte N. (2012). Small organometallic compounds as antibacterial agents. Dalton Trans..

[24] Ma D.L., Wang M., Mao Z., Yang C., Ng C.T., Leung C.H. (2016). Rhodium complexes as therapeutic agents. Dalton Trans..

[25] Chandra S., Tyagi M., Agrawal S. (2011). Spectral and antimicrobial studies on tetraaza macrocyclic complexes of PdII, PtII, RhIII and IrIII metal ions. J. Saudi Chem. Soc..

[26] Beloglazkina E.K., Manzheliy E.A., Moiseeva A.A., Maloshitskaya O.A., Zyk N.V., Skvortsov D.A., Osterman I.A., Sergiev P.V., Dontsova O.A., Ivanenkov Y.A. (2016). Synthesis, characterisation, cytotoxicity and antibacterial activity of ruthenium(II) and rhodium(III) complexes with sulfur-containing terpyridines. Polyhedron.

[27] Fandzloch M., Augustyniak A.W., Dobrzanska L., Jedrzejewski T., Sitkowski J., Wypij M., Golinska P. (2020). First dinuclear rhodium(II) complexes with triazolopyrimidines and the prospect of their potential biological use. J. Inorg. Biochem..

[28] Aradhyula B.P.R., Joshi N., Poluri K.M., Kollipara M.R. (2018). Synthesis and antibacterial studies of rhodium and iridium complexes comprising of dipyridyl hydrazones. J. Mol. Struct..

[29] Mansouri G., Heidarizadi F., Naghipour A., Notash B. (2016). Synthesis, characterization and antibacterial study of cyclometalated rhodium(III) complex containing dithiocarbamate. J. Mol. Struct..

[30] Vaidya M., McBain A.J., Banks C.E., Whitehead K.A. (2019). Single and combined antimicrobial efficacies for nine metal ion solutions against *Klebsiella pneumoniae*, Acinetobacter baumannii and Enterococcus faecium. Int. Biodeterior. Biodegrad..

[31] Dizaj S.M., Lotfipour F., Barzegar-Jalali M., Zarrintan M.H., Adibkia K. (2014). Antimicrobial activity of the metals and metal oxide nanoparticles. Mater. Sci. Eng. C.

[32] Ott I. (2020). Metal N-heterocyclic carbene complexes in medicinal chemistry. Adv. Inorg. Chem..

[33] Bernier C.M., DuChane C.M., Martinez J.S., Falkinham J.O., Merola J.S. (2021). Synthesis, Characterization, and Antimicrobial Activity of RhIII and IrIII N-Heterocyclic Carbene Piano-Stool Complexes. Organometallics.

[34] Simpson P.V., Schmidt C., Ott I., Bruhn H., Schatzschneider U. (2013). Synthesis, cellular uptake and biological activity against pathogenic microorganisms and cancer cells of rhodium and iridium N-heterocyclic carbene complexes bearing charged substituents. Eur. J. Inorg. Chem..

[35] Streciwilk W., Terenzi A., Cheng X., Hager L., Dabiri Y., Prochnow P., Bandow J.E., Wolfl S., Keppler B.K., Ott I. (2018). Fluorescent organometallic rhodium(I) and ruthenium(II) metallodrugs with 4-ethylthio-1,8-naphthalimide ligands: Antiproliferative effects, cellular uptake and DNA-interaction. Eur. J. Med. Chem..

[36] Cetinkaya B., Cetinkaya E., Küçükbay H., Durmaz R. (1996). Antimicrobial activity of carbene complexes of rhodium(I) and ruthenium(II). Arzneimittelforschung.

[37] Wang J., Sánchez-Roselló M., Aceña J.L., Del Pozo C., Sorochinsky A.E., Fustero S., Soloshonok V.A., Liu H. (2014). Fluorine in pharmaceutical industry: Fluorine-containing drugs introduced to the market in the last decade (2001–2011). Chem. Rev..

[38] Zhou Y., Wang J., Gu Z., Wang S., Zhu W., Acenã J.L., Soloshonok V.A., Izawa K., Liu H. (2016). Next Generation of Fluorine-Containing Pharmaceuticals, Compounds Currently in Phase II-III Clinical Trials of Major Pharmaceutical Companies: New Structural Trends and Therapeutic Areas. Chem. Rev..

[39] Isanbor C., O’Hagan D. (2006). Fluorine in medicinal chemistry: A review of anti-cancer agents. J. Fluorine Chem..

[40] Kirk K.L. (2006). Fluorine in medicinal chemistry: Recent therapeutic applications of fluorinated small molecules. J. Fluorine Chem..

[41] Müller K., Faeh C., Diederich F. (2007). Fluorine in pharmaceuticals: Looking beyond intuition. Science.

[42] Rufino-Felipe E., Colorado-Peralta R., Reyes-Márquez V., Valdés H., Morales-Morales D. (2021). Fluorinated-NHC transition metal complexes: Leading characters as potential anticancer metallodrugs. Anti-Cancer Agents Med. Chem..

[43] Turcio-García L.Á., Valdés H., Hernández-Ortega S., Canseco-Gonzalez D., Morales-Morales D. (2022). Arylation of aldehydes catalyzed by fluorinated NHC–Rh(I) complexes. New J. Chem..

[44] Turcio-García L.Á., Valdés H., Arenaza-Corona A., Hernández-Ortega S., Morales-Morales D. (2023). Electronic properties and supramolecular study of selenoureas with fluorinated-NHC ligands derived from imidazo [1,5-a]pyridines. New J. Chem..

[45] Yang H., Lou C., Sun L., Li J., Cai Y., Wang Z., Li W., Liu G., Tang Y. (2019). admetSAR 2.0: Web-service for prediction and optimization of chemical ADMET properties. Bioinformatics.

[46] SwissADME. http://www.swissadme.ch/index.php#/.

