# Supplementary material for: Fluorinated Rh(I)–NHC Compounds as Potential Antibacterials Against Multidrug-Resistant Klebsiella pneumoniae Clinical Isolates Producing ESBL
Source: Pharmaceutics. 2025 Jul 28;17(8):973. doi: 10.3390/pharmaceutics17080973 (PMC12389553; doi:10.3390/pharmaceutics17080973)
Supplement: Supplementary file 1 [file pharmaceutics-17-00973-s001.zip › pharmaceutics-3717614-SI-Corrected.pdf]

Supporting information

Fluorinated Rh(I)–NHC compounds as potential antibacterials against multidrug-resistant Klebsiella pneumoniae clinical isolates producing ESBL.

Luis Ángel Turcio-García[1], Ricardo Parra-Unda[2,*], Hugo Valdés[3], Simón Hernández-Ortega[1], Gladymar Guadalupe Valenzuela-Ramirez[2], Yesmi Patricia Ahumada-Santos[2], Yesenia Sánchez-Lugo[2], Viviana Reyes-Marquez[4], David Morales-Morales[1,*].

[1]Instituto de Química, Universidad Nacional Autónoma de México, Circuito Exterior, Ciudad Universitaria, Coyoacán, Ciudad de México C.P. 04510, México. [2]Unidad de Investigaciones en Salud Pública, Facultad de Ciencias Químico Biológicas, Universidad Autónoma de Sinaloa, Culiacán 80013, Sinaloa, México. [3]Departamento de Química Orgánica y Química Inorgánica, Instituto de Investigación Química "Andrés M. del Río" (IQAR), Facultad de Farmacia, Universidad de Alcalá, Alcalá de Henares, 28805 Madrid, Spain. [4]Departamento de Ciencias Químico-Biológicas, Universidad de Sonora, Luis Encinas y Rosales S/N, Hermosillo C.P. 83000, Sonora, México.

1. Materials and methods 2
   1.1. Isolation and characterization of Klebsiella pneumoniae 2
   1.2. Antibacterial activity evaluation (MIC/MBC) 2
   1.3. Time–killing kinetics of Rh(I)–NHC complexes 3
   1.4. ADMET prediction/Pharmacokinetic parameters 4
2. X–ray structures and crystallographic data 4
   2.1. Data collection and refinement for compound Rh–1, Rh–6 and Rh–8 4
   2.2. Compound Rh–1 5
   2.3. Compound Rh–6 6
   2.4. Compound Rh–8 7
3. References 8

1. Materials and methods

The synthesis and characterization of the azolium salts and complexes were conducted in accordance with our previously published method.[1-2] All reactions were carried out under a nitrogen atmosphere using standard Schlenk techniques, unless otherwise noted. All chemical compounds were commercially obtained from Aldrich Chemical Co. and used without further purification. The ^{1}H and $^{13}C\{^{1}H\}$ NMR spectra were obtained using a Bruker Ascend 500 spectrometer or a JEOL GX300 spectrometer. The chemical shifts were reported in ppm downfield of TMS, using the residual signals in the solvent as the internal standard. Elemental analyses were performed on a Perkin Elmer 240. MS–Electrospray determinations were recorded on a Bruker Daltonics–Esquire 3000 plus Electrospray Mass Spectrometer. Mass measurements in FAB+ were performed at a resolution of 3000 using magnetic field scans, with the matrix ions as the reference material, or alternatively, by electric field scans with the sample peak bracketed by two reference ions (polyethylene glycol or cesium iodide).

1.1. Isolation and characterization of Klebsiella pneumoniae

Fifteen clinical isolates of Klebsiella pneumoniae were collected from the Department of Teaching at the General Hospital of Culiacán, Sinaloa, between February 2019 and 2020. These isolates were identified using the VITEK automated system (bioMérieux, Inc., Hazelwood, MO, United States), and their morphology and colony characteristics were determined on McConkey agar. Gram staining and recommended biochemical tests, including the urease test, lysine test, indole test, citrate test from Simmons, ornithine test, and mobility test, were performed for structural identification (cell shape and wall). The isolates were collected following the necessary approvals from the ethics and research committee of the hospital for the present study.

We conducted antibacterial tests using strains obtained from the American Type Culture Collection (ATCC), including *Staphylococcus aureus* ATCC 25923, 29213, and ATCC 43300, *Escherichia coli* ATCC 25922, *Enterococcus faecalis* ATCC 29212, and *Klebsiella pneumoniae* ATCC 700603, in accordance with the protocols recommended by the Institute of Clinical and Laboratory Standards.[3] Additionally, we included a strain of Streptococcus pneumoniae that was isolated from an oropharyngeal exudate. Furthermore, we used six strains of multi–resistant Klebsiella pneumoniae producing ESBL that were obtained from patients who were treated at a public hospital in Culiacán, Sinaloa, Mexico, and were collected from different types of samples.

1.2. Antibacterial activity evaluation (MIC/MBC)

The compounds were analyzed quantitatively in broth microdilution tests to determine the lowest concentration to inhibit bacterial growth (MIC) completely in a range of concentrations of 0.06–250 µg/mL. First, a series of dilutions was prepared at twice the desired concentration to be evaluated, from a stock solution at a concentration of 500 µg/mL by filling a 96–well microplate in triplicate with 50 µL of the compounds. Then, 50 µL of bacterial solution containing 10^{6} CFU/mL was added. Linezolid, Gentamicin (0.125–32 µg/mL) and Amoxicillin (4–256 µg/mL) were used as positive controls, while negative controls were inoculum without antibiotic or compound. The compounds were dissolved with DMSO (10%) without any detectable effect on bacterial growth. The microplate was incubated at 37°C for 18–20 h.

We used control strains from the ATCC (29213, 25923, 43300, 25922, 29212, and 700603) as well as an isolate of *Streptococcus spp*. Minimum inhibitory concentrations (MICs) were determined using the Institute of Clinical Laboratory Standards parameters to ensure quality control and methodological reliability. The MIC values fell within the cut–off points for determining antibiotic susceptibility.

The MIC value was established as the minimum concentration at which no turbidity or growth spurt was observed in the well. In addition, the minimum bactericidal concentration (MBC) was determined by subculturing the assay dilution of microtiter plate wells where no turbidity was observed from the MIC in McConkey medium and incubating at 37 °C for 18 – 20 hours. The highest dilution that did not produce bacterial colonies was taken as MBC.

1.3. Time–killing kinetics of Rh(I)–NHC complexes

To determine the bactericidal kinetics of the organometallic compounds, we employed the broth macrodilution method. For this analysis, we selected the compounds with the lowest MIC values, as determined by the broth microdilution method. Specifically, we generated curves for **Rh–1**, which was active against 9 strains, **Rh–6**, which was active against 7 strains, and **Rh–7**, which was active against 2 strains. The MIC values for these compounds ranged from 3.9 to 250 µg/mL.

The isolates were cultured in Mueller Hinton Broth (MHB). To prepare the compounds, falcon tubes were filled with 10 mL of Mueller Hinton medium, and the indicated solvent (DMSO) was added to dissolve the compounds. After the compounds were completely dissolved, they were added to the falcon tubes containing the Mueller Hinton Broth and stirred. Next, 20 µL of the inoculum, previously adjusted to 0.5 of the McFarland standard (1x108 CFU/mL with absorbance of 0.08 at 625nm), was added to the falcon tubes. The tubes were then incubated at 37°C with gentle shaking for 24 hours, and aliquots were taken at each seeding time (0, 2, 4, 6, and 24 hours).

The inoculum was processed in Eppendorf tubes, as follows: 300 µL of the inoculum was centrifuged at 13000 RPM for 2–3 minutes, the supernatant was decanted, and the pellet was resuspended in 300 µL of saline solution. From the previous preparation, 300 µL of the resuspended inoculum was added to row A of a 96–well microplate to obtain an approximate inoculum concentration of $5x10^5$ CFU/mL. Then, 270 µL of saline was added to each well from row B down. Using a multichannel pipette, 30 µL of the suspension from row A was transferred to row B, mixed by pipetting three times, and 30 µL of this mixture was transferred to row C. This process was repeated until the dilutions were completed ($5x10^5$, $5x10^4$, $5x10^3$, $5x10^2$, $5x10^1$, $5x10^0$, $5x10^{-1}$ and $5x10^{-2}$). Next, 10 µL of each dilution was transferred to an MHA plate to form a line of approximately 4 to 5 cm. The plates were then incubated at 37ºC for 18–20 hours, and the CFU/mL were counted using an automatic bacterial colony counter (UVP Colony Doc–It Imaging Station Fisher Scientific), considering the dilution factor. The experiments were conducted in triplicate and the results

were used to generate graphs of log CFU/mL versus time (0–24h). Bactericidal activity was defined as a reduction of ≥3 log10 CFU/mL relative to the initial inoculum, while bacteriostatic activity was defined as a decrease of <3 log10 CFU/mL relative to the initial inoculum.

To assess the efficacy of **Rh–1**, two concentrations (1X MIC (62.5 µg/mL) and 4X MIC (250 µg/mL) were used against *S. aureus* (ATCC 29213), with linezolid as the positive control. Similarly, the clinical isolate of *Klebsiella pneumoniae* (U–13815) was treated with a concentration of 1X MIC of **Rh–1** and **Rh–6**, with Amoxicillin used as the control antibiotic. In addition, the bactericidal kinetics of **Rh–7** against *S. aureus* (ATCC 29213) and *E. coli* (ATCC 25922) were evaluated using 1X MIC, with gentamicin serving as the positive control.

Subsequently, a Gram stain test was performed to visualize the concentration of CFU of *K. pneumoniae* (U–13815) after exposure to the compounds **Rh–1** and **Rh–6**, compared to the positive control (Amoxicillin).

1.4. ADMET prediction/Pharmacokinetic parameters

The prediction of absorption, distribution, metabolism, excretion, and toxicity (ADMET) properties is crucial in drug design and development. We used the admetSAR (http://lmmd.ecust.edu.cn/admetsar1/) to calculate parameters such as blood–brain barrier penetration, human intestinal absorption (HIA), human oral bioavailability, Caco–2 permeability, AMES mutagenesis, carcinogenicity, and aquatic toxicity. In addition, we utilized the SwissADME website (http://www.swissadme.ch/) to calculate physicochemical descriptors and predict ADMET parameters, pharmacokinetic properties, drug–likeness, and chemical compatibility of the compounds. The following parameters were evaluated: water solubility (Log S) SILICOS–IT, gastrointestinal absorption, similarity to Lipinski's rule of five, synthetic accessibility, and lead resemblance[4-5]

2. X–ray structures and crystallographic data

2.1. Data collection and refinement for compound Rh–1, Rh–6 and Rh–8

Yellow prism crystals of **Rh–1** (CCDC 2280627), **Rh–6** (CCDC 2280628) and **Rh–8** (CCDC 2280629) complexes were obtained by growing them from CH_2Cl_2/hexane and mounted them on glass fibers. The crystals were then analyzed using a Bruker D8 adventure κ geometry diffractometer equipped with a micro–focus X–ray source of Mo–target X–ray source (λ=0.71073 Å). The detector was placed 5.0 cm away from the crystals, and frames were collected with a scan width of 0.3 in ω and exposure time of 5 s/frame. The Bruker SAINT software package was used to collect and integrate 66261 reflections using a narrow-frame integration algorithm.[6] The structures were solved using Patterson methods with the SHELXS–2014/7 program.[7] The remaining atoms were located *via* a few cycles of least squares refinements and difference Fourier maps. Hydrogen atoms were input at calculated positions and allowed to ride on the atoms to which they are attached. Thermal parameters were refined for all hydrogen atoms using a Ueq = 1.2 Å. The final cycle of refinement was

carried out on all non–zero data using SHELXL–2014/7.[7] Absorption correction was applied using SADABS program.[8]

2.2. Compound Rh–1

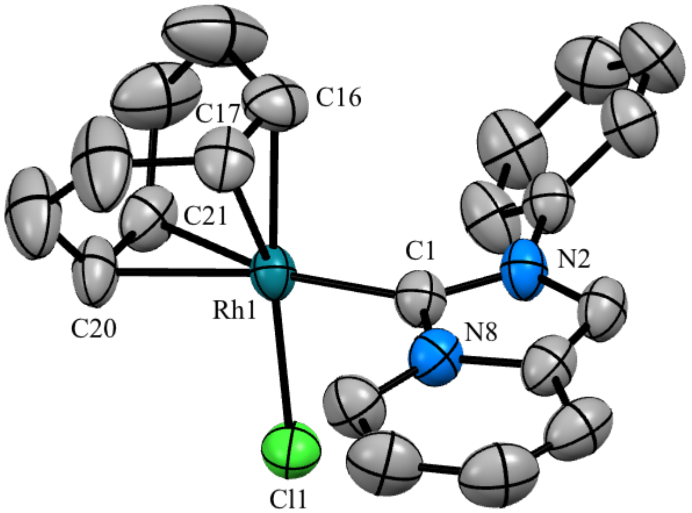

Figure S1. Molecular structure of complex **Rh–1**

Table S1. Crystal data and structure refinement for (**Rh–1)**

	**(Rh–1)**
Empirical formula	$C_{43}H_{48}Cl_2N_4ORh_2$
Formula weight	913.57
Temperature/K	298(2) K
Crystal system	Triclinic
Space group	P-1
a/Å	11.4616(6)
b/Å	12.5504(6)
c/Å	14.6797(7)
α/°	85.0618(14)
β/°	80.4555(13)
γ/°	70.5153(13)
Volume/Å^3	1962.08(17)
Z	2
ρ_{calc}g/cm^3	1.546
μ/mm^{-1}	1.016
F(000)	932
Crystal size/mm^3	0.388 x 0.297 x 0.242
Radiation	MoKα (λ = 0.71073)
2Θ range for data collection/°	4.376 to 50.848
Index ranges	-10<=h<=13, -14<=k<=15, -17<=l<=17
Reflections collected	22826

Independent reflections	7210 [R(int) = 0.0223]
Data/restraints/parameters	7210 / 2 / 473
Goodness–of–fit on F^2	1.101
Final R indexes [I>=2σ (I)]	R1 = 0.0326, wR2 = 0.0811
Final R indexes [all data]	R1 = 0.0376, wR2 = 0.0865
Largest diff. peak/hole/e $Å^{-3}$	0.609/-0.498

2.3. Compound Rh–6

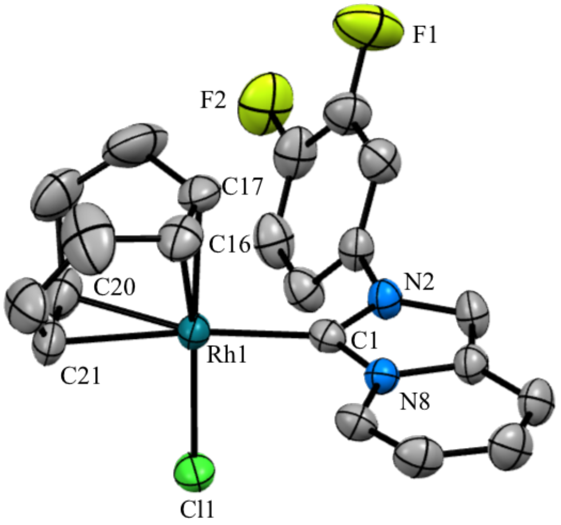

Figure S2. Molecular structure of complex **Rh–6**

Table S2. Crystal data and structure refinement for (**Rh–6)**

	**(Rh–6)**
Empirical formula	$C_{21}H_{19}ClF_2N_2Rh$
Formula weight	475.74
Temperature/K	279(2)
Crystal system	Triclinic
Space group	P-1
a/Å	7.7651(3)
b/Å	8.4262(3)
c/Å	14.4635(5)
α/°	93.3040(10)
β/°	92.6070(10)
γ/°	92.7100(10)
Volume/$Å^3$	942.62(6)
Z	2
ρ_{calc}g/cm^3	1.676
μ/mm^{-1}	1.074
F(000)	478
Crystal size/mm^3	0.339 x 0.210 x 0.120

Radiation	MoKα (λ = 0.71073)
2Θ range for data collection/°	4.848 to 50.754
Index ranges	-9<=h<=7, -10<=k<=10, -17<=l<=17
Reflections collected	11139
Independent reflections	3451 [R(int) = 0.0219]
Data/restraints/parameters	3451 / 0 / 244
Goodness–of–fit on F^2	1.144
Final R indexes [I>=2σ (I)]	R1 = 0.0322, wR2 = 0.0713
Final R indexes [all data]	R1 = 0.0369, wR2 = 0.0744
Largest diff. peak/hole/e $Å^{-3}$	0.594/-0.376

2.4. Compound Rh–8

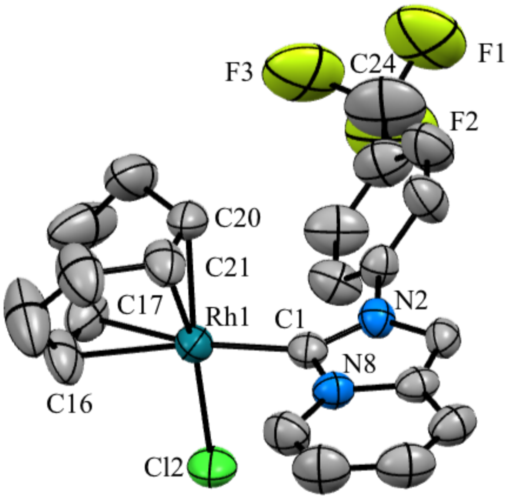

Figure S3. Molecular structure of complex **Rh–8**

Table S3. Crystal data and structure refinement for (**Rh–8)**

	**(Rh–8)**
Empirical formula	$C_{45}H_{43}Cl_5F_6N_4Rh_2$
Formula weight	1136.90
Temperature/K	298(2)
Crystal system	Orthorhombic
Space group	Pbca
a/Å	14.0090(5)
b/Å	19.2486(6)
c/Å	34.2218(13)
α/°	90
β/°	90
γ/°	90
Volume/$Å^3$	9228.0(6)

Z	8
ρ_{calc}g/cm^3	1.637
μ/mm^{-1}	1.066
F(000)	4560.0
Crystal size/mm^3	0.279 × 0.242 × 0.164
Radiation	MoKα (λ = 0.71073)
2Θ range for data collection/°	4.312 to 50.802
Index ranges	$-16 \le h \le 16$, $-23 \le k \le 23$, $-40 \le l \le 41$
Reflections collected	86408
Independent reflections	8466 [Rint = 0.0836, Rsigma = 0.0460]
Data/restraints/parameters	8466/255/643
Goodness–of–fit on F^2	1.065
Final R indexes [I>=2σ (I)]	R1 = 0.0522, wR2 = 0.1068
Final R indexes [all data]	R1 = 0.0952, wR2 = 0.1246
Largest diff. peak/hole/e Å^{-3}	0.735/-0.413

3. References

[1] L. Á. Turcio-García, H. Valdés, S. Hernández-Ortega, D. Canseco-Gonzalez, D. Morales-Morales, *New J. Chem.* **2022**, *46*, 16789-16800.

[2] L. Á. Turcio-García, H. Valdés, A. Arenaza-Corona, S. Hernández-Ortega, D. Morales-Morales, *New J. Chem.* **2023**, *47*(4), 2090-2095.

[3] F. Li, M. Feterl, J. M. Warner, A. I. Day, F. R. Keene, J. G. Collins, *Dalton Trans.* **2013**, *42*(24), 8868-8877.

[4] admetSAR, http://lmmd.ecust.edu.cn/admetsar2/ **2020**.

[5] SwissADME, http://www.swissadme.ch/index.php#/, **2020**.

[6] *Bruker 2018. Programas: APEX3, SAINT, Bruker AXS Inc., Madison, Wisconsin, USA.*

[7] G. Sheldrick, *Acta Cryst. C* **2015**, *71*(1), 3-8.

[8] L. Krause, R. Herbst-Irmer, G. M. Sheldrick, D. Stalke, *J. Appl. Crystallogr.* **2015**, *48*(1), 3-10.
